# Supplementary material for: Genome-Wide Association Study for Root Morphology and Phosphorus Acquisition Efficiency in Diverse Maize Panels
Source: Int J Mol Sci. 2023 Mar 25;24(7):6233. doi: 10.3390/ijms24076233 (PMC10094163; doi:10.3390/ijms24076233)
Supplement: Supplementary file 1 [file ijms-24-06233-s001.zip › SupplementaryMaterials.pdf]

Supplementary Materials

Supplementary Tables

Table S1. A complete list of the maize lines including their pedigree and origin (Excel).

Table S2. Significant SNPs identified through MLM analysis based on a -log (P-value) ≥ 5 for the traits average of root diameter (RD, cm), root length (RL, cm), root surface area (SA, cm^2), total seedling dry weight (TDW, mg) and total P content (PCont, mg) under low and high P concentration and functional annotation of candidate genes (GO terms). (Excel)

Supplementary Figures

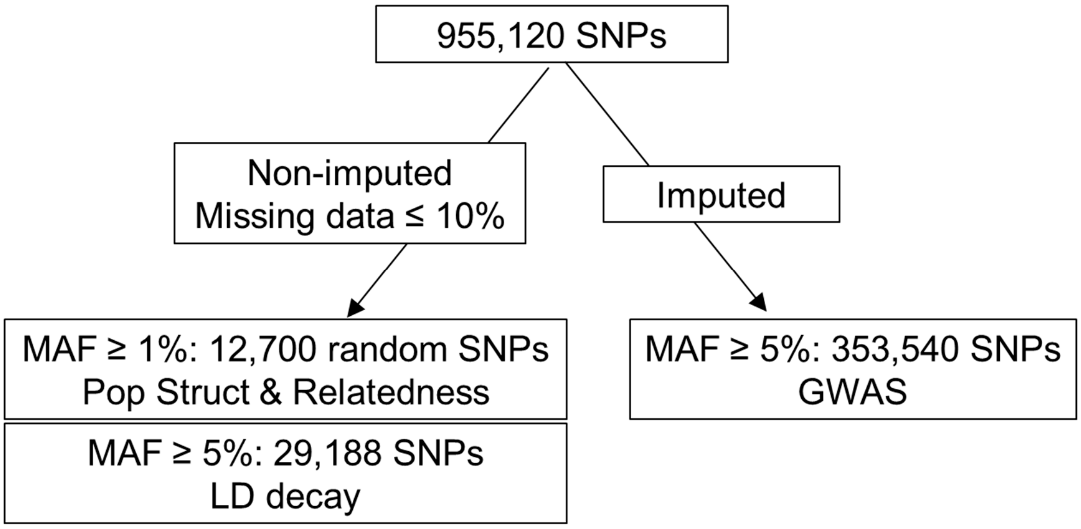

Figure S1. A schematic flowchart of the GBS-based SNP filtering process used for GWAS, population structure, familial relatedness and LD decay.

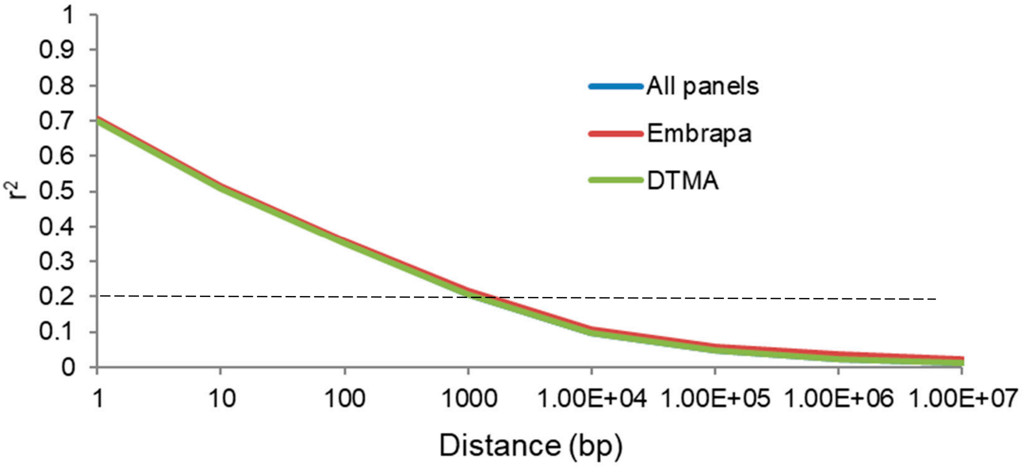

Figure S2. Linkage disequilibrium decay measured as squared genotypic correlations between pair of SNPs (r^2) in function of physical distance in base pairs (bp) based on 29,188 SNPs. The maize lines were considered within all panels, and within Embrapa and DTMA panels separately. Dashed lines represent the threshold of 0.2.

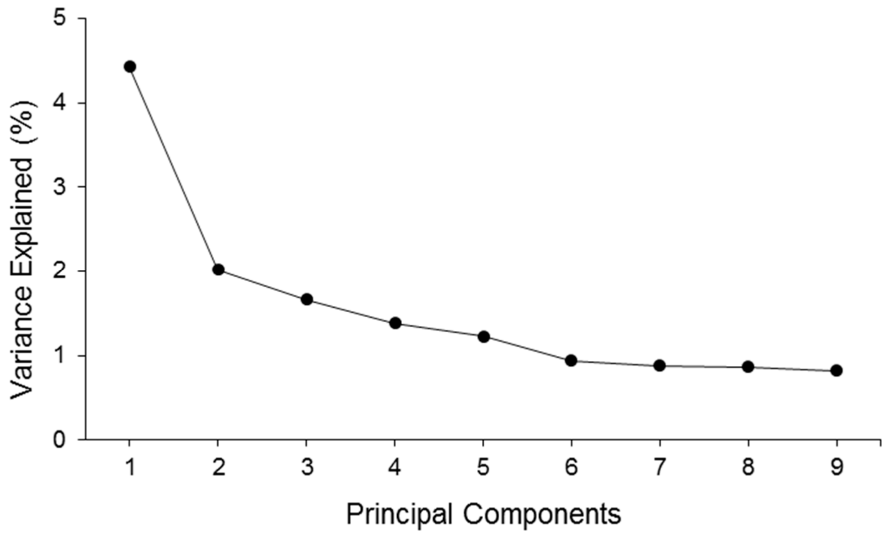

Figure S3. Percentage of total variance explained by each principal component using 31,790 GBS-based SNPs in a panel of 561 maize lines.

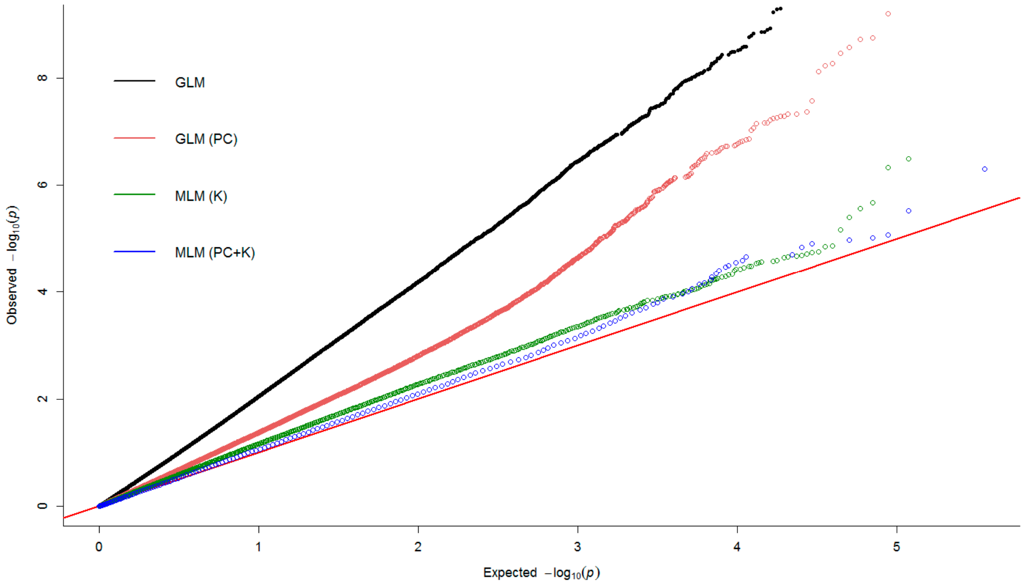

Figure S4. Q-Q plot for root length under low P conditions using naïve, PC, K and PC+K in a panel of 561 tropical maize inbred lines with a total of 353,540 SNP markers.

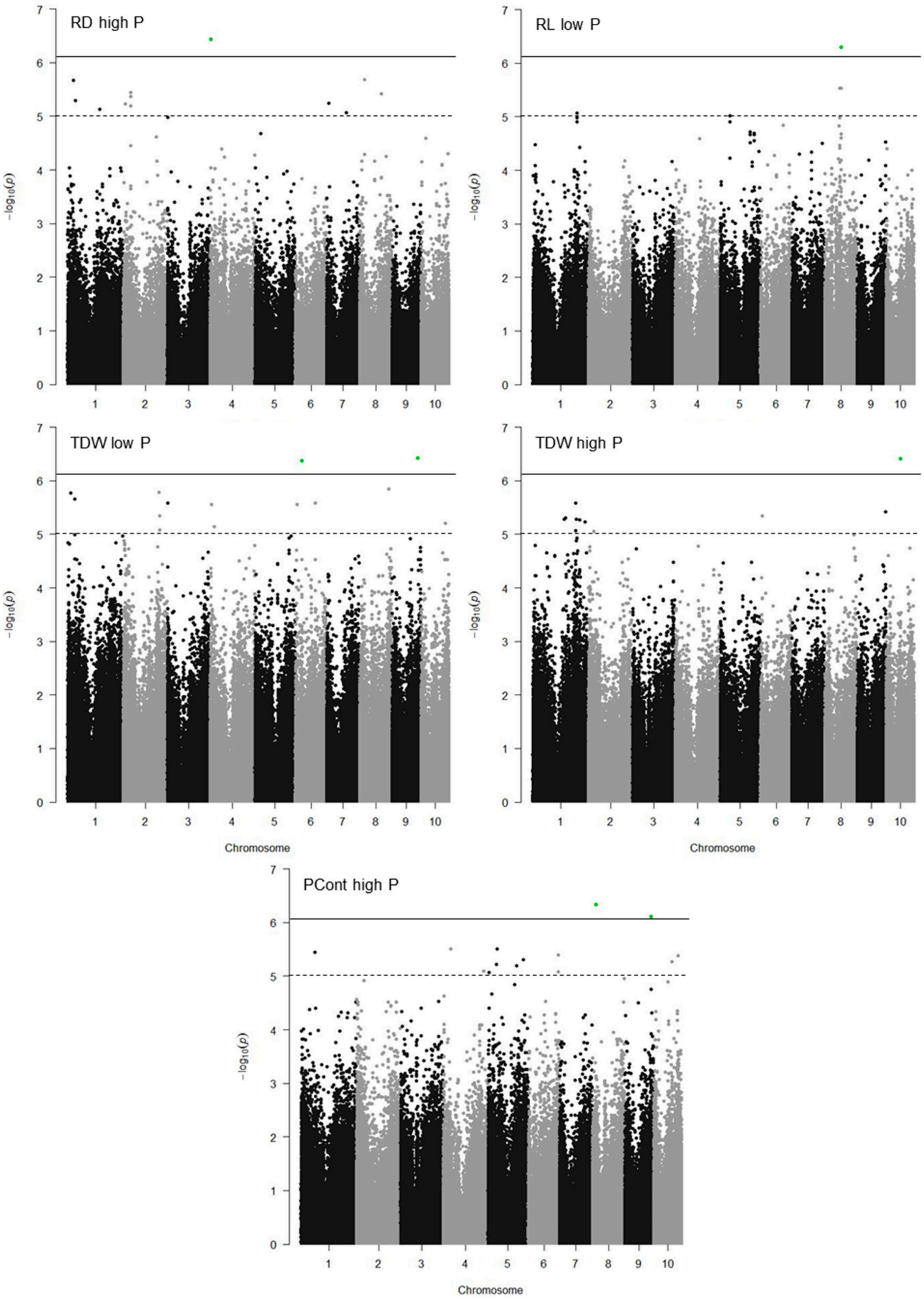

Figure S5. Manhattan plot for phenotypic traits that showed significant SNPs at -$\log_{10}$(*p*-value) ≥ 6.07. The association analyses used MLM (PC + K) model in a panel of 561 tropical maize lines using 353,540 SNP markers for the traits average of root diameter in mm (RD), root length in cm (RL), total seedling dry weight in mg (TDW) and total P content in mg (PCont).

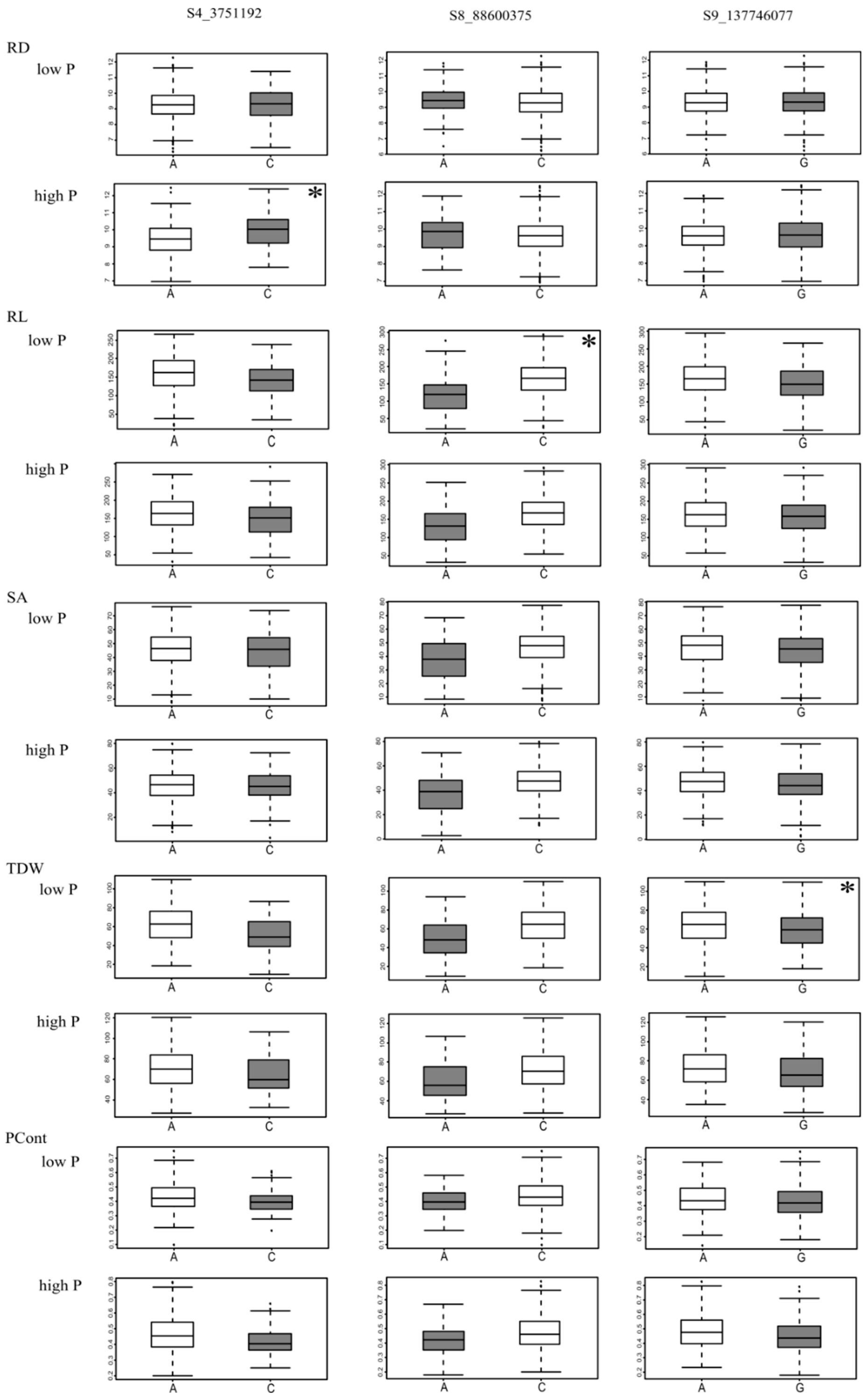

Figure S6. Boxplot of the SNPs S4_3751192, S8_88600375 and S9_137746077 that are associated with average of root diameter (RD), root length (RL), root surface area (SA), total seedling dry weight (TDW) and total P content (PCont) under low and high P conditions. The asterisk represents significant associations at $\log_{10}(p\text{-value}) \geq 6.07$. The white boxes show the favorable allele for enhancement of P acquisition.
